# Characteristics of Pathogens of Acute Exacerbation of Chronic Obstructive Pulmonary Disease Hospitalized Patients: A Retrospective Study From 2010 to 2020

**DOI:** 10.1155/pm/4025205

**Published:** 2025-11-11

**Authors:** Songsong Yu, Tiecheng Yang

**Affiliations:** Department of Emergency, Emergency and Critical Care Medical Center, Beijing Shijitan Hospital, Capital Medical University, Beijing, China

**Keywords:** acute exacerbation, chronic obstructive pulmonary disease, drug susceptibility, hospitalization, pathogenic bacteria

## Abstract

**Aim:**

The aim of the study is to evaluate the distribution and drug resistance of infectious pathogens in hospitalized patients with acute exacerbation of chronic obstructive pulmonary disease (AECOPD).

**Methods:**

This is a retrospective study of AECOPD patients who underwent pathogen evaluation and drug susceptibility tests.

**Results:**

A total of 199 hospitalized AECOPD patients were analyzed. Among them, 77.9% had monoinfection, and 22.1% had multiple infections. Two hundred and eighty-eight strains were isolated, with 61.1% gram-negative, 3.8% gram-positive, and 35.0% fungi, while 58 strains were colonized. Common bacteria included *Haemophilus parainfluenzae*, *Acinetobacter baumannii*, and *Pseudomonas aeruginosa*. *Staphylococcus aureus* and *Enterococcus faecalis* were the main gram-positive cocci, and *Pseudohyphae* were the main fungi. Fifty gram-negative strains showed drug resistance (19 colonized strains), with high resistance to ceftriaxone in *A. baumannii*, *P. aeruginosa*, *Klebsiella pneumoniae*, and *Escherichia coli*. Methicillin-resistant *S. aureus* was resistant to penicillin but sensitive to other antibiotics.

**Conclusion:**

The study highlights the distribution of pathogens and the prevalence of drug-resistant strains among AECOPD patients.

## 1. Introduction

According to the Global Initiative for Chronic Obstructive Lung Disease (GOLD) report in 2023, chronic obstructive pulmonary disease (COPD) is defined as a heterogeneous lung condition characterized by chronic respiratory symptoms due to abnormalities of the airways and/or alveoli that cause persistent, often progressive, airflow obstruction [1]. COPD presents a significant global health challenge, affecting individuals primarily over 40 years old. COPD represents a major cause of morbidity, mortality, and healthcare use worldwide [2]. In the United States, 12% (30 million) adults developed COPD and this disease ranks as the fourth leading cause of death [3]. In addition, COPD accounts for more than 3% of all physician office visits annually, thus leading to substantial economic burden on healthcare systems and society, encompassing both direct medical costs and indirect costs [3]. Acute exacerbation of chronic obstructive pulmonary disease (AECOPD) is a major cause of hospital admissions and can significantly impact the quality of life for individuals with COPD [4–6]. AECOPD is often triggered by respiratory infections, environmental pollutants, or other factors that cause inflammation and narrowing of the airways [7–9]. AECOPD leads to increased breathlessness, coughing, mucus production, and reduced lung function [10, 11]. It can be severe and require hospitalization, as it poses a significant risk to patients with compromised lung function.

With regret, numerous patients with COPD experience exacerbations even when diligently adhering to the therapeutic strategies. These acute exacerbations result in worsened symptoms, a reduction in lung function, and an elevated mortality rate [12]. A recent report suggested that in patients with AECOPD, about half of them had pathogen infection [13]. Feng et al. conducted a multicenter study and found that atypical pathogens play an important role in AECOPD [14]. In addition, Yoo et al. suggested that bacterial prevalence in patients with moderate AECOPD in South Korea showed correlations with the global prevalence, without regional differences [15]. Therefore, antibiotics are frequently prescribed for AECOPD treatment, since bacterial infections often contribute to exacerbations. However, overuse, inappropriate prescription, and incomplete courses of antibiotics can lead to the development of antibiotic resistance [16, 17]. Patients with resistant infections often require more potent antibiotics, and the risk of recurrent exacerbations and poor clinical outcomes also escalates.

Therefore, comprehending the prevalence and drug resistance profiles of pathogens causing infections in hospitalized individuals with AECOPD, as well as making informed choices regarding antimicrobial agents, holds paramount importance for patient outcomes. This study was aimed at evaluating the etiological distribution and drug resistance of pathogens in hospitalized patients with AECOPD.

## 2. Patients and Methods

### 2.1. Study Design and Subjects

This retrospective study included hospitalized patients with AECOPD who underwent pathogen detection and drug susceptibility tests in the Department of Respiratory Medicine at Beijing Shijitan Hospital, Capital Medical University, between January 2010 and December 2020. Inclusion criteria are as follows: (1) patients with AECOPD who were diagnosed according to the Guidelines for the Diagnosis and Treatment of Chronic Obstructive Pulmonary Disease (2013 revised edition) [18] and (2) positive sputum specimen culture. Exclusion criteria are as follows: (1) patients with bronchiectasis (medical record homepage diagnosed or had a previous history), interstitial fibrosis, active tuberculosis, lung cancer, or other lung diseases such as acute pulmonary embolism and (2) incomplete data. For patients who were repeatedly admitted to the hospital due to AECOPD, only the clinical data from the first hospitalization were collected. Determination of bacterial infection status includes the following: (1) patients presenting with purulent sputum, (2) white blood cell or neutrophil counts or CRP increased, (3) exudation on chest CT scan, and any two of the three conditions were met.

Identification of patients with COPD includes the following: (1) patients diagnosed as COPD before admission based on lung function test during stable stage and (2) patients diagnosed as COPD in discharge based on lung function test after symptom of acute exacerbation controlled during hospitalization.

This study was approved by the Ethics Committee of Beijing Shijitan Hospital, Capital Medical University (Approval Number 2018(66); Date: Nov. 8, 2018). Due to the study's nature of the retrospective design, written informed consent was waived.

### 2.2. Data Collection

Clinical data including age, gender, smoking history, disease severity, ICU admission, mechanical ventilation, hospital stay duration, respiratory failure, survival status, and results for sputum specimen culture were collected.

#### 2.2.1. Typical Procedures for Sputum Specimen Collection and Culture

Sputum specimens were collected after the patients' admission to the hospital. The sampling protocol involved rinsing the mouth three times in the morning, followed by deep coughing to obtain sputum specimens. These specimens were sent for examination over 3 consecutive days posthospitalization. Pathogen identification consisted of inoculating specimens onto blood agar plates and China blue plates (incubated at 37°C for 18–72 h). Bacterial identification and drug susceptibility were conducted using Mérieux's Vitek-II system. If needed, the K-B paper diffusion analysis was employed for additional drug susceptibility tests. Cefoxitin resistance was utilized to identify methicillin-resistant *Staphylococcus aureus* (MRSA). These procedures followed the standards outlined by the American Committee for Clinical Laboratory Standards (CLSI M100 edition) and were conducted in accordance with the National Clinical Laboratory Procedures. Mérieux's Vitek-II system was also used for extended-spectrum *β*-lactamase (ESBL) examination, including bacterial identification and drug sensitivity testing. Pathogenic bacteria identification criteria were as follows: (1) presence of the same dominant bacteria in sputum cultures for more than 2 consecutive days; (2) bacterial concentration ≥ 10^7^ cfu/mL; and (3) exclusion of *Streptococcus*, *Neisseria putida*, yeasts, diphtheria bacillus, and other bacteria associated with respiratory colonization.

### 2.3. Statistical Analysis

Statistical analysis was conducted using SPSS 22.0 (IBM, United States). Only descriptive analysis was used in this study. Data are presented as number (percentage) or mean (standard deviation, SD).

## 3. Results

### 3.1. Baseline Characteristics

A total of 199 AECOPD patients who had positive sputum specimens were included for analysis. Their mean age was 75.91 ± 9.51 years old, and 142 (71.4%) were male. Among them, 155 (77.9%) had monoinfection and 44 (22.1%) had multiple infections. Thirty-nine patients were admitted to the ICU; the mean hospital stay duration was 15.16 (7–16) days (Table 1).

### 3.2. Pathogen Distribution

A total of 288 strains of pathogenic bacteria were isolated, including 176 strains of gram-negative bacilli (61.1%), 11 strains of gram-positive cocci (3.8%), and 101 strains of fungi (35.0%). Among the identified gram-negative bacilli, the most common were *Haemophilus parainfluenzae*, *Acinetobacter baumannii*, *Pseudomonas aeruginosa*, *Stenotrophomonas maltophilia*, and *Klebsiella pneumoniae* subspecies *pneumoniae*. *S. aureus* and *Enterococcus faecalis* were the predominant gram-positive cocci, while *Pseudohyphae* were the predominant fungi (Table 2).

Among bacterial strains, 37 strains were considered colonized and were not treated with corresponding anti-infection therapy, including *P. aeruginosa* nine strains, *A. baumannii* 10 strains, *H. parainfluenzae* nine strains, *Haemophilus influenzae* one strain, and MRSA eight strains. Among fungal strains, 21 strains were considered colonized and were not treated with corresponding antifungal therapy, including *Candida albicans* seven strains, *Torulopsis glabrata* three strains, *Candida tropicalis* two strains, *Candida parapsilosis* one strain, *Aspergillus glaucus* four strains, *Aspergillus fumigatus* two strains, *Aspergillus flavus* one strain, and *Aspergillus niger* one strain.

### 3.3. Drug Sensitivity

Among the identified gram-negative bacilli, 50 strains (28.4%) presented with drug resistance with 19 colonized strains including *A. baumannii* (52.0%, 61.5% infected strain), *P. aeruginosa* (18.0%, none of them infected strain), *K. pneumoniae* (8.0%, all were infected strains), *Escherichia coli* (6.0%, all were infected strains), and *A. baumannii*/*Haemolyticus immobilis* (6.0%, all infected strains). Nine strains (90.9%) of gram-positive cocci with drug resistance were present; all of them were MRSA, but only one of them was an infected strain (Table 3). Drug susceptibility tests showed that the resistance rate of *A. baumannii*, *P. aeruginosa*, *K. pneumoniae* subspecies *pneumoniae*, and *E. coli* to ceftriaxone was up to 90%, and the resistance rates of the first three bacteria (*A. baumannii*, *P. aeruginosa*, and *K. pneumoniae* subspecies *pneumoniae*) to imipenem were higher than 75%. *K. pneumoniae* subspecies *pneumoniae* was strongly resistant to antibiotics, and *E. coli* was sensitive to imipenem, piperacillin tazobactam sodium, and cefoperazone sulbactam. MRSA was resistant to penicillin, but all were sensitive to vancomycin, linezolid, cotrimoxazole and tigecycline (Table 4).

## 4. Discussion

The present study included 199 AECOPD patients who had positive sputum specimen cultures and found that 288 strains of pathogens were isolated, while 58 strains were colonized, and most of them were gram-negative bacilli. In addition, the resistance rate of *A. baumannii*, *P. aeruginosa*, *K. pneumoniae* subspecies *pneumoniae*, and *E. coli* to ceftriaxone was up to 90%. MRSA was resistant to penicillin but sensitive to vancomycin, linezolid, cotrimoxazole, and tigecycline. These findings underscore the significant prevalence of bacterial strains and fungi among AECOPD patients and the high-rate drug-resistant pathogens in this cohort.

With the widespread use of broad-spectrum antibiotics, aging, increasing incidence, and prolonged hospitalization, the pathogenic distribution in AECOPD patients is changing. A previous study in Spain revealed that the most frequent pathogens isolated from sputum were *P. aeruginosa*, *H. influenzae*, *Streptococcus pneumoniae*, *Moraxella catarrhalis*, and *S. aureus* [19]. In Sweden, AECOPD patients requiring ICU admission showed high gram-negative respiratory isolates, including *Pseudomonas* and *Enterobacteriaceae* spp. [20]. In mainland China, the predominant bacteria for AECOPD were *P. aeruginosa*, *K. pneumoniae*, *H. influenzae*, *S. pneumoniae*, *H. parainfluenzae*, *A. baumannii*, *M. catarrhalis*, and *E. coli* [21]. In another study in Kunming, Yunnan, China, the most common bacterial flora were *S. pneumoniae*, *P. aeruginosa*, *H. influenzae*, *M. catarrhalis*, and *K. pneumoniae* [22]. Lieberman et al. found that in most AECOPD patients, serological evidence suggested acute *Chlamydia pneumoniae* infection around the time of the exacerbation [23], and *Mycoplasma pneumoniae* infection is common in AECOPD patients [24]. A recent Chinese study identified the atypical pathogen distribution of hospitalized AECOPD patients and found that *M. pneumoniae*, *C. pneumoniae*, and *Legionella pneumophila* were common in clinics [14]. In addition, Lieberman et al. also showed that about a quarter of hospitalized AECOPD patients had at least one classic bacterial etiology: *S. pneumoniae*, *H. influenzae*, and *M. catarrhalis* [25]. In South Korea, Yoo et al. found that the most commonly isolated pathogen was *H. influenzae*, followed by *S. pneumoniae* and *P. aeruginosa* in moderate exacerbation of COPD patients [15]. *P. aeruginosa*, *K. pneumoniae*, and *A. baumannii* were the Top 3 potentially pathogenic microorganisms in the early readmission AECOPD patients [26]. Moreover, the AECOPD patients had high gram-negative bacteria and Enterobacteriaceae infections [27]. In this study, sputum culture results of hospitalized AECOPD patients revealed a predominance of gram-negative bacillus and fungal infections. In addition, the most frequently isolated pathogens were gram-negative bacilli (*H. parainfluenzae*, *A. baumannii*, *P. aeruginosa*, *S. maltophilia*, and *K. pneumoniae* subspecies *pneumonia*), fungi (*Pseudohyphae*), and gram-positive cocci (*S. aureus* and *E. faecalis*). Based on these findings, it could be seen that gram-negative bacilli were frequently isolated in AECOPD patients, but the specific bacteria slightly differed. For instance, we and others found that *H. parainfluenzae*, *A. baumannii*, *P. aeruginosa*, and *K. pneumoniae* were common. However, *M. catarrhalis* was not isolated from this cohort of patients, but fungi were identified. This discrepancy may be attributed to the following: (1) This cohort of patients was old aged and had prolonged illness, low immune function, and poor nutritional status, making them more susceptible to fungal infections. (2) The majority of patients included were individuals with severe COPD who were admitted due to recurrent acute exacerbations. Upon hospitalization for acute exacerbation, the prolonged empirical use of broad-spectrum antibiotic therapy increased the likelihood of fungal infections. In addition, these patients underwent frequent antibiotic and glucocorticoid treatments, altering the bacterial composition. (3) The use of inhaled glucocorticoid therapy. Despite gargling and deep sputum specimen collection prior to retention, the potential for contamination and oral bacterial colonization reaching the lower respiratory tract remained. (4) Some patients underwent systemic glucocorticoid treatment, leading to suppressed immune function and significantly elevated risk of fungal infections. (5) Regional and epidemiological traits of the pathogenic bacteria. Notably, this led to the emergence of drug-resistant strains. Future prospective studies with large sample sizes are needed to validate the findings.

In this study, drug sensitivity test revealed that *H. influenzae* was sensitive to *β*-lactamase antibiotics. However, *A. baumannii*, *P. aeruginosa*, *K. pneumoniae*, and *E. coli* displayed varying degrees of resistance to *β*-lactamase, cephalosporin, and quinolone antibiotics. Notably, the resistance rate to ceftriaxone reached up to 90% and imipenem exhibiting the highest level of antibiotic resistance. In contrast to a study in China, isolated bacterial pathogens were susceptible to imipenem [21], which indicated the antibiotic resistance shift in the last 10 decades. *S. aureus* has shown resistance to *β*-lactamases, levofloxacin, and imipenem while demonstrating no resistance to vancomycin and linezolid, which is in line with previous findings [28–30]. For cases involving severe COPD patients with recurring hospitalizations, in situations where the effectiveness of active broad-spectrum antibiotic treatment proves unsatisfactory [31], a heightened vigilance is essential to detect potential drug-resistant gram-positive cocci infections. In such scenarios, prompt administration of vancomycin and linezolid for anti-infective treatment may be recommended.

This study is not without limitations. Its retrospective nature coupled with a restricted sample size could potentially impact the generalizability of the findings. Furthermore, due to the study's design, the presence of recall bias cannot be discounted. Additionally, this study did not include atypical bacterial and viral infections. Notably, prior studies indicated a significant prevalence of viral and atypical infections among AECOPD patients [14, 32, 33]. Finally, this study was retrospective and we could not detect the bacterial load for each pathogen, so we determined whether the patient was infected status through symptoms, laboratory results, and chest imaging manifestations. Future endeavors should focus on well-structured prospective studies, incorporating substantial sample sizes, to comprehensively monitor pathogen distribution and drug resistance profiles among hospitalized AECOPD patients.

## 5. Conclusion

This study sheds light on the distribution of bacterial strains and fungi among AECOPD patients during hospitalization, along with the prevalence of drug-resistant pathogens within this cohort. These findings underscore the pressing need for implementing robust infection control measures. Further study is warranted to validate these findings.

## Figures and Tables

**Table 1:** Demographic characteristics.

**Variable**	**Number (%)/mean (SD)**
Age	75.91 ± 9.53
Gender	
Male	142 (71.4%)
Female	57 (28.6%)
Smoking history	
Yes	146 (73.4%)
No	53 (26.6%)
Disease severity	
Severe and above	132 (66.3%)
Respiratory failure	
Type I	22 (11.1%)
Type II	61 (30.7%)
ICU admission	39 (19.6%)
Mechanical ventilation	
Noninvasive	31 (15.6%)
Invasive	12 (6.0%)
Pathogen infection	
Monoinfection	155 (77.9%)
Multiple infection	44 (22.1%)
Hospital stay duration (days)	15.16 (7–16)
Survival status	
Dead	11 (5.5%)

**Table 2:** Pathogen distribution.

	**Number (infected)**	**% (infected)**
Gram-negative bacilli	176 (147)	61.1% (63.9%)
*Haemophilus parainfluenzae*	55 (46)	31.3% (31.3%)
*Acinetobacter baumannii*	33 (23)	18.8% (15.6%)
*Pseudomonas aeruginosa*	16 (7)	9.1% (4.8%)
*Stenotrophomonas maltophilia*	15 (15)	8.5% (10.2%)
*Haemophilus influenzae*	11 (11)	6.3% (7.5%)
*Klebsiella pneumoniae*	11 (11)	6.3% (7.5%)
*Klebsiella pneumoniae* subspecies *pneumoniae*	10 (10)	5.7% (6.8%)
*Klebsiella pneumoniae* subspecies *ozaenae*	1 (1)	0.6% (0.7%)
*Acinetobacter baumannii/haemolyticus*	5 (5)	2.8% (3.4%)
*Escherichia coli*	4 (4)	2.3% (2.7%)
*Enterobacter cloacae*	4 (4)	2.3% (2.7%)
*Klebsiella acidophilus*	4 (4)	2.3% (2.7%)
*Fusobacterium roqueforti*	3 (3)	1.7% (2.0%)
Xylose-oxidizing alkaline-producing bacilli subspecies xylo	2 (2)	1.1% (1.4%)
*Proteus mirabilis*	2 (2)	1.1% (1.4%)
Other	11 (11)	6.3% (7.5%)
Gram-positive cocci	11 (3)	3.8% (1.3%)
*Staphylococcus aureus*	10 (2)	90.9% (66.7%)
*Enterococcus faecalis*	1 (1)	9.1% (33.3%)
Fungus	101 (80)	35.0% (34.8%)
*Candida*	78 (65)	77.2% (81.3%)
*Candida albicans*	45 (38)	57.7% (58.5%)
*Candida tropicalis*	10 (10)	12.8% (15.4%)
*Torulopsis glabrata*	8 (5)	10.3% (7.7%)
*Candida glabrata*	7 (5)	9.0% (7.7%)
*Candida krusei*	2 (2)	2.6% (3.1%)
Others	6 (5)	7.7% (7.7%)
*Aspergillus*	23 (15)	22.8% (18.8%)
*Aspergillus fumigatus*	12 (10)	52.2% (66.7%)
*Aspergillus glaucus*	5 (1)	21.7% (6.7%)
*Aspergillus flavus*	4 (3)	17.4% (20%)
*Aspergillus niger*	2 (1)	8.7% (6.7%)

**Table 3:** Drug susceptibility for gram-negative bacilli.

	***Acinetobacter baumannii* (** **n** = 26**)**					***Pseudomonas aeruginosa* (** **n** = 9**)**					***Klebsiella pneumoniae* (** **n** = 4** )**					***Escherichia coli* (** **n** = 3**)**				
	**Resistant**	**Intermediate**	**Sensitive**	**Not available**	**Resistance rate**	**Resistant**	**Intermediate**	**Sensitive**	**Not available**	**Resistance rate**	**Resistant**	**Intermediate**	**Sensitive**	**Not available**	**Resistance rate**	**Resistant**	**Intermediate**	**Sensitive**	**Not available**	**Resistance rate**
Ampicillin/sulbactam	19	3	2	2	79.2%	3	0	1	5	75.0%	4	0	0	0	100%	3	0	0	0	100%
Cefepime	21	2	2	1	84.0%	3	5	1	0	33.3%	3	0	1	0	75.0%	3	0	0	0	100%
Ceftazidime	23	1	2	0	88.5%	5	1	3	0	55.6%	4	0	0	0	100%	2	0	1	0	66.7%
Ceftriaxone	24	2	0	0	92.3%	5	0	0	4	100%	4	0	0	0	100%	3	0	0	0	100%
Ciprofloxacin	24	0	2	0	92.3%	9	0	0	0	100%	4	0	0	0	100%	2	0	1	0	66.7%
Levofloxacin	16	3	7	0	61.5%	8	0	1	0	88.9%	3	0	1	0	75.0%	3	0	0	0	100%
Piperacillin/tazobactam	14	2	2	8	77.8%	6	1	2	0	66.7%	3	0	1	0	75.0%	0	1	2	0	0%
Cefoperazone/sulbactam	2	6	11	7	10.5%	4	1	4	0	44.4%	2	1	0	1	66.7%	0	1	1	1	0%
Imipenem	23	3	0	0	88.5%	7	0	2	0	77.8%	3	0	1	0	75.0%	0	0	3	0	0%
Cotrimoxazole	15	0	11	0	57.7%	4	0	0	5	100%	3	1	0	0	75.0%	0	1	1	1	0%

**Table 4:** Drug susceptibility for gram-positive cocci.

	**Methicillin-resistant *Staphylococcus aureus* (** **n** = 9**)**				
	**Resistant**	**Intermediate**	**Sensitive**	**Not available**	**Resistance rate**
Penicillin	9	0	0	0	100%
Ciprofloxacin	9	0	0	0	100%
Levofloxacin	7	0	0	2	100%
Clindamycin	5	0	4	0	55.6%
Moxifloxacin	7	1	0	1	87.5%
Imipenem	3	0	0	6	100%
Vancomycin	0	0	9	0	0%
Linezolid	0	0	9	0	0%
Cotrimoxazole	0	0	8	1	0%
Tigecycline	0	0	5	4	0%

## Data Availability

The authors confirm that the data supporting the findings of this study are available within the article.
